# Pro-Apoptotic Function Analysis of the Reaper Homologue IBM1 in *Spodoptera frugiperda*

**DOI:** 10.3390/ijms21082729

**Published:** 2020-04-15

**Authors:** Benshui Shu, Jingjing Zhang, Sethuraman Veeran, Guohua Zhong

**Affiliations:** 1 Guangzhou City Key Laboratory of Subtropical Fruit Trees Outbreak Control, Zhongkai University of Agriculture and Engineering, Guangzhou 510225, China; shubenshui@126.com; 2 Key Laboratory of Crop Integrated Pest Management in South China, Ministry of Agriculture and Rural Affairs, South China Agricultural University, Guangzhou 510642, China; zhangjingjing@stu.scau.edu.cn (J.Z.); sethuramanbio@gmail.com (S.V.); 3 Key Laboratory of Natural Pesticide and Chemical Biology, Ministry of Education, South China Agricultural University, Guangzhou 510642, China

**Keywords:** *Spodoptera frugiperda*, apoptosis, Sf-IBM1, overexpression, mitochondria

## Abstract

As an important type of programmed cell death, apoptosis plays a critical role in lepidopteran insects in response to various internal and external stresses. It is controlled by a network of genes such as those encoding the inhibitor of apoptosis proteins. However, there are few studies on apoptosis-related genes in *Spodoptera frugiperda*. In this study, an orthologue to the *Drosophila* reaper gene, named *Sf-IBM1*, was identified from *S. frugiperda*, and a full-length sequence was obtained by reverse transcription polymerase chain reaction (RT-PCR) and rapid amplification of cDNA ends PCR (RACE-PCR). The expression pattern of *Sf-IBM1* was determined in different developmental stages and various tissues. Apoptotic stimuli including azadirachtin, camptothecin, and ultraviolet radiation (UV) induced the expression of Sf-IBM1 at both transcript and protein levels. Overexpression of *Sf-IBM1* induced apoptosis in Sf9 cells, and the Sf-IBM1 protein was localized in mitochondria. The apoptosis induced by Sf-IBM1 could be blocked by the caspase universal inhibitor carbobenzoxy-valyl-alanyl-aspartyl-[*O*-methyl]-fluoromethylketone (Z-VAD-FMK) and Sf-IAP1. Our results provide valuable information that should contribute to a better understanding of the molecular events that lead to apoptosis in lepidopterans.

## 1. Introduction

Apoptosis is a highly conserved cellular process in metazoans and is responsible for eliminating supernumerary, deleterious, or defective cells. Apoptosis is also crucial for maintaining tissue homeostasis and normal development as well as responding to cytotoxic stress [1,2]. Apoptosis can be initiated via two highly conserved signal transduction pathways, the intrinsic mitochondrial pathway and the external death receptor pathway, both of which lead to the eventual activation of caspases [3]. Caspases are cysteine-aspartic proteases and can be divided into initiator caspases and effector caspases that existed as inactive monomers in cells [4,5]. Upon apoptotic stimuli, initiator caspase caspase-9 (Dronc in *Drosophila*) is recruited by the adaptor protein Apaf-1 (Dark in *Drosophila*) through the amino-terminal caspase recruitment domain (CARD) to form an apoptosome [6]. The initiator caspases form dimers and then are autocleaved in an apoptosome, resulting in activated initiator caspases that can then activate downstream effector caspases, again through proteolytical cleavage. Activated effector caspases can cleave the cellular components, leading to eventual apoptosis [7].

Apoptosis is inhibited by the inhibitor of apoptosis proteins (IAPs), which are endogenous caspase inhibitors and were originally identified in the genomes of lepidopteran baculoviruses [8]. Numerous IAPs have been identified from various insects and have been demonstrated to be the negative regulators of apoptosis [9]. For example, the *Drosophila* IAP1 (DIAP1) can enhance ubiquitylation and inhibit the activity of Dronc both in vitro and in vivo [10]. Co-expression of *Bm-IAP1* in *Bombyx mori* suppresses apoptosis induced by overexpression of *Bm-Dronc* in BM-N cells [9]. IAPs contain two functional domains, the baculoviral IAP repeat (BIR) domain and the Really Interesting New Gene (RING) domain. The IBR domain may be involved in direct inhibition of apoptosis, whereas the RING domain may take part in protein–protein interactions. IAPs can bind to both initiator and effector caspases directly and degrade activated caspases through the E3 ubiquitin ligase activity, resulting in inhibition of apoptosis [11].

The anti-apoptotic function of IAPs can be neutralized by IAP antagonists. IAP antagonists are the proteins containing the evolutionarily conserved IAP binding motif (IBM), which consists of several amino acids at the N-terminal [12]. In *Drosophila*, five RHG (Rpr, Hid, Grim) family proteins including Reaper, Hid, Grim, Sickle, and Jafrac2 have been identified, each with an IBM motif in the N-terminus and identified as the IAP antagonists [13]. Similarly, two IAP antagonists, Smac/Diablo and HtrA2/Omi have also been identified in mammals [14]. IAP antagonists compete with caspases by binding to the BIR domains of IAPs with different affinities via IBM directly [15]. Besides, the IAP antagonists can also function as positive regulators of apoptosis by inducing the auto-ubiquitylation of DIAP1 in *Drosophila* [16].

The IAP antagonist Reaper from *Drosophila* has been studied relatively extensively. Reaper can bind to both BIR1 and BIR2 domains of IAPs through IBM [17]. Reaper can also be recruited and interact with Hid via the Grim_helix3 (GH3) domain, resulting in mitochondrial localization and promotion of auto-ubiquitylation and subsequent degradation of DIAP1 [18,19]. Homologs of Reaper from several other insects have also been studied. For example, two IAP antagonists Michelob_x (Mx) and IMP have been characterized in *Aedes aegypti* and have been found to act as pro-apoptotic factors that compete with caspases for binding to AeIAP1 [15]. The expression of Mx can also be induced by baculovirus infection in larval midgut cells [20]. Strong induction of Reaper has been observed in embryos of *Anastrepha suspense* following γ-irradiation treatments. Functional synergy between *As-hid* and *As-rpr* has been reported in *A. suspense* as well [2,21]. In lepidopterans, only one Reaper homolog has been identified and named as IBM1 in *B. mori*. Function analysis has indicated that IBM1 acts as a pro-apoptotic protein and can interact with BmIAP1 and BmNPV IAP2 [13,22].

*Spodoptera frugiperda* is a serious lepidopteran pest worldwide. Very limited information is available on the machinery of apoptosis in this insect. A gene encoding an inhibitor of apoptosis protein, named *Sf-IAP*, has been identified and characterized. Sf-IAP inhibits apoptosis by inhibiting the activity of Sf-caspase-1 [23]. No IAP antagonist has been identified, and the molecular basis for apoptosis remains unclear in *S. frugiperda*. In this study, a Reaper homolog, named *Sf-IBM1*, was cloned and characterized. The expression profiles of *Sf-IBM1* were examined via qRT-PCR in different developmental stages and tissues. The expression patterns of *Sf-IBM1* in Sf9 cells treated with different apoptotic stimuli were also examined using both RT-qPCR and western blots. We found that overexpression of *Sf-IBM1* induced apoptosis in Sf9 cells by activating the mitochondrial apoptosis pathway, and apoptosis was inhibited by the caspase general inhibitor carbobenzoxy-valyl-alanyl-aspartyl-[*O*-methyl]-fluoromethylketone (Z-VAD-FMK) completely. Co-expression of *Sf-IAP* inhibited apoptosis. Our results indicate that Sf-IBM1 plays a pro-apoptotic role in Sf9 cells and has functional similarity to an RHG family protein in *Drosophila*.

## 2. Results

### 2.1. Cloning and Sequencing Sf-IBM1

**Figure 1:**
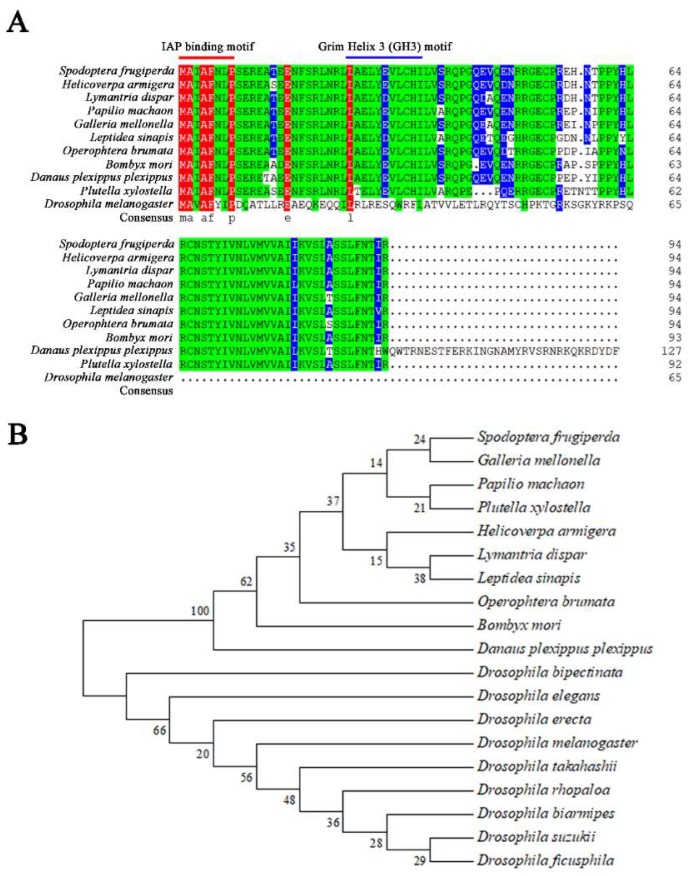
Sequence similarity among IBM1s from different insects. (**A**) A multiple sequence alignment of Sf-IBM1 together with IBM1s from other insects. GeneBank accession numbers of IBM1s were shown as the following: *Helicoverpa armigera* Ha-IBM1: PZC80231.1; *Lymantria dispar* Ld-IBM1: BAW32728.1; *Papilio machaon* Pm-IBM1: XP_014360463.1; *Galleria mellonella* Gm-IBM1: XP_026755064.1, *Leptidea sinapis* Ls-IBM1: VVC93562.1; *Operophtera brumata* Ob-IBM1: KOB72267.1; *Bombyx mori* Bm-IBM1: NP_001159813.1; *Danaus plexippus plexippus* Dp-IBM1: OWR53643.1, *Plutella xylostella* Px-IBM1: AHL68668.1 and *Drosophila melanogaster* Reaper: NP_524138.1; The red and green bases in the figure indicate highly conserved regions, while the blue bases indicate moderately conserved regions, and white bases indicate non-conserved regions. (**B**) A Phylogenic tree of Sf-IBM1 together with homologous proteins from other insect species.

To explore whether a gene homologous to RHG exists in *S. frugiperda*, we searched a transcriptome of Sf9 cells and identified a truncate unigene annotated as the Reaper homolog IBM1. To obtain a full-length cDNA of *Sf-IBM1*, the 3′ and the 5′ untranslated regions of the transcript were obtained via rapid-amplification of cDNA ends polymerase chain reaction (RACE-PCR). An 890 bp transcript was cloned, which contains the full-length coding region plus 154 bp at 5′- and 451 bp at 3′ untranslated regions. The predicted coding sequences (CDS) encodes a protein with 94 amino acid residues and a predicted molecular weight of 10.81 kDa. No signal peptide was found in the predicted protein. A transmembrane domain was predicted, spanning from residue 69 to 91 (TYIVNLVMVVAIIKVSLASSLFN). Multiple sequence alignments by DNAMAN software showed two highly conserved motifs, the IAP binding motif consisting of the seven amino acid residues MAIAFNL at the N-terminus and the Grim Helix 3 (GH3) motif consisting of the 13 amino acid residues LNRLLAELYEVLCHI. Sf-IBM1 shares more than 90% amino acid sequence identify with IBM1s from other lepidopteran insects, including *Lymantria dispar*, *Operophtera brumata*, *B. mori*, *Danaus plexippus plexippus*, and *Plutella xylostella*. However, little sequence similarity was found to the Reaper from *D. melanogaster* except for the IAP binding motif (Figure 1A). A phylogenetic tree of IBM1s constructed by neighbor-joining also revealed a closer evolutionary relationship among the proteins from lepidopterans (Figure 1B).

### 2.2. Expression Patterns of Sf-IBM1 among Different Developmental Stages and Tissues

**Figure 2:**
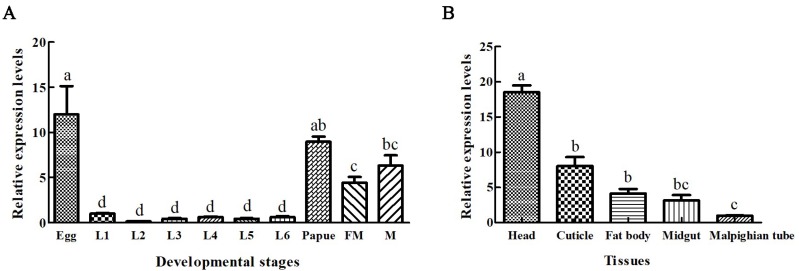
The transcript abundance of *Sf-IBM1* in insects at different developmental stages and in tissues of sixth instar larvae. (**A**) The transcript abundance of *Sf-IBM1* in insects at different developmental stages. L: larvae, FM: female, M: male; (**B**) The expression pattern of *Sf-IBM1* in various tissues. *Sf-GAPDH* was used as the reference gene for qRT-PCR results normalization. Different letters above the bars show significant differences between different samples (*p* < 0.05).

The expression levels of *Sf-IBM1* in whole insects at different developmental stages and in different tissues of sixth instar larvae were determined using qRT-PCR. *Sf-IBM1* was expressed throughout the developmental stages but with significantly higher expression levels in eggs, pupae, and adults (Figure 2A). *Sf-IBM1* was detected in various tissues, but the expression levels showed great variation among different tissues. The head exhibited the highest expression followed by cuticles, fat bodies, and midguts. The Malpighian tubules showed the lowest expression level (Figure 2B).

### 2.3. Apoptotic Stimuli Induced the Up-Regulation of Sf-IBM1

**Figure 3:**
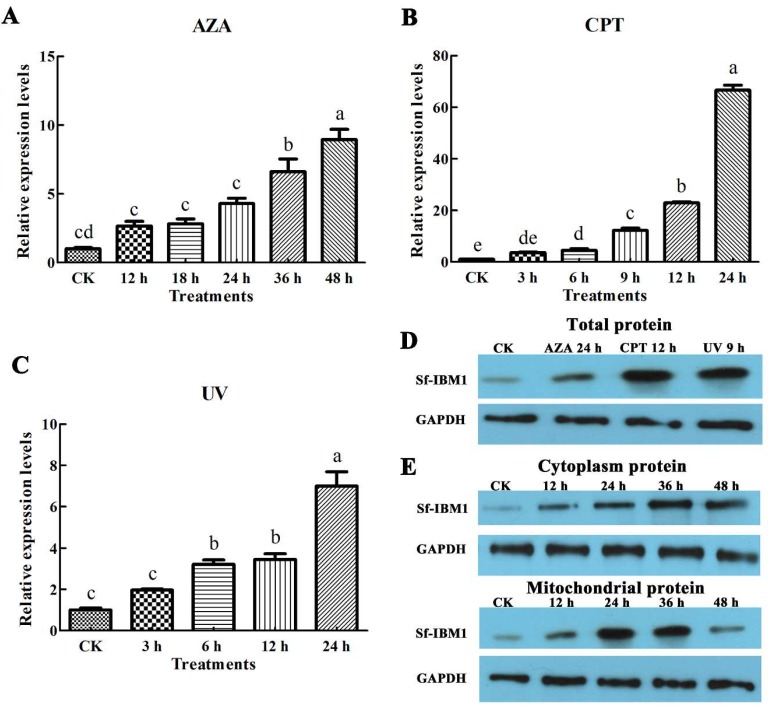
Apoptotic stimuli increased the expression of Sf-IBM1 in Sf9 cells. (**A**) Changes in *Sf-IBM1* transcript abundance in Sf9 cells treated with 0.75 μg/mL azadirachtin for different times. (**B**) Changes in *Sf-IBM1* transcript abundance in Sf9 cells treated with 1.0 μg/mL camptothecin for different times. (**C**) Changes of *Sf-IBM1* transcript abundance in Sf9 cells exposed to UV for 5 min and recovered for different times. (**D**) Changes of Sf-IBM1 protein abundance in Sf9 cells treated with apoptotic stimuli. (**E**) Sf-IBM1 protein distribution between cytoplasm and mitochondria in Sf9 cells treated with 0.75 μg/mL azadirachtin for different times. Different letters above bars show significance of differences between different samples (*p* < 0.05). AZA: azadirachtin; CPT: camptothecin.

To determine whether apoptosis affected the expression of *Sf-IBM1*, Sf9 cells were exposed to three apoptotic stimuli including azadirachtin, camptothecin, and UV. As shown in Figure 3, all the three apoptotic stimuli increased the expression of *Sf-IBM1* in a time-dependent manner. Compared with control cells, the transcript levels of *Sf-IBM1* increased 2.61-, 2.77-, 4.23-, 6.46-, and 8.88-fold when cells were treated with azadirachtin for 12, 18, 24, 36, and 48 h, respectively (Figure 3A). Camptothecin also affected the transcript levels of *Sf-IBM1* significantly, which increased to 3.64-, 4.42-, 12.17-, 22.9-, and 66.56-fold in cells treated for 3, 6, 9, 12, and 24 h, respectively (Figure 3B). UV caused 1.97-, 3.20-, 3.44-, and 6.97-fold increases in *Sf-IBM1* transcript abundance in cells exposed to UV for 5 min followed by recovering for 3, 6, 9, and 12 h, respectively (Figure 3C).

Protein levels in cells treated with different apoptotic stimuli were analyzed via western blots. The protein abundance of Sf-IBM1 was up-regulated after the cells were treated with azadirachtin for 24 h, camptothecin for 12 h, and UV irradiation (Figure 3D), which supported the transcript level results that apoptotic stimuli that acted on Sf9 cells could increase the expression of Sf-IBM1. To further analyze Sf-IBM1 protein levels in different cellular fractions, cytoplasic and mitochondrial proteins were extracted from cells treated with azadirachtin for different times and analyzed via western blots. As shown in Figure 3E, Sf-IBM1 in cytoplasm increased after azadirachtin treatments in a time-dependent manner. At the same time, this protein in mitochondria was also up-regulated following azadirachtin treatments. The protein level in mitochondria was highest at 24 h after azadirachtin treatments, followed by a gradual decrease over time. These results suggest that azadirachtin up-regulated Sf-IBM1 protein expression in both cytoplasm and mitochondria of Sf9 cells.

### 2.4. Transient Expression of Sf-IBM1 Induced Apoptosis in Sf9 Cells

**Figure 4:**
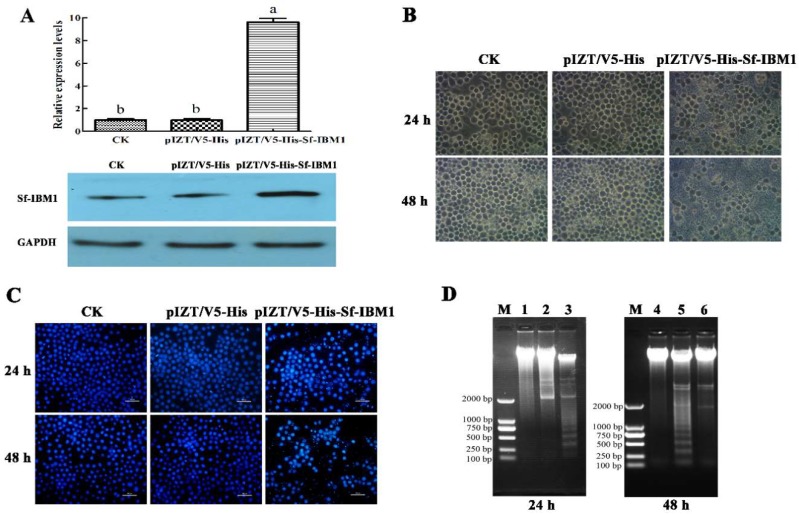
Overexpression of Sf-IBM1 induced apoptosis in Sf9 cells. (**A**) Transfection with pIZT/V5-His-Sf-IBM1 increased transcriptional and protein levels of Sf-IBM1 in Sf9 cells. (**B**) Apoptosis induced by transfection with pIZT/V5-His-Sf-IBM1 in Sf9 cells was observed by an inverted phase-contrast microscope (40×). (**C**) The nuclear morphological changes in Sf9 cells overexpressed Sf-IBM1 based on DAPI staining observed under a fluorescence microscopy (20×). (**D**) DNA ladders in samples obtained from Sf9 cells transfected with pIZT/V5-His-Sf-IBM1. M: DL2000 marker; 1 and 4: DNA samples isolated from normal cells; 2 and 6: DNA samples extracted from cells transfected with the control vector pIZT/V5-His; 3 and 5: DNA samples extracted from cells transfected with pIZT/V5-His-Sf-IBM1.

To explore the role of Sf-IBM1 in apoptosis, the construct pIZT/V5-His-Sf-IBM1 was made to express Sf-IBM1 in Sf9 cells. Sf-IBM1 increased in cells transfected with pIZT/V5-His-Sf-IBM1 at both transcript and protein levels compared to controls (Figure 4A). Apoptosis was induced following transient expression of *Sf-IBM1* in Sf9 cells based on three criteria. First, apoptosis was detected on an inverted phase-contrast microscope. Apoptotic bodies were widely distributed in cells transfected with pIZT/V5-His-Sf-IBM1 for 24 and 48 h (Figure 4B). Second, 4’,6-diamidino-2-phenylindole (DAPI) staining revealed irregular and agglutinated nuclei in pIZT/V5-His-Sf-IBM1-transfected cells, which was accompanied with the increased nucleosomes and nucleosomic fragments (Figure 4C). Third, DNA ladders characteristic of apoptosis were observed in cells transfected with pIZT/V5-His-Sf-IBM1 for 24 and 48 h but could not be found in control cells and cells transfected with pIZT/V5-His (Figure 4D). These observations demonstrated that Sf-IBM1 is a positive regulator of apoptosis in Sf9 cells.

### 2.5. Sf-IBM1 Localized to Mitochondria

**Figure 5:**
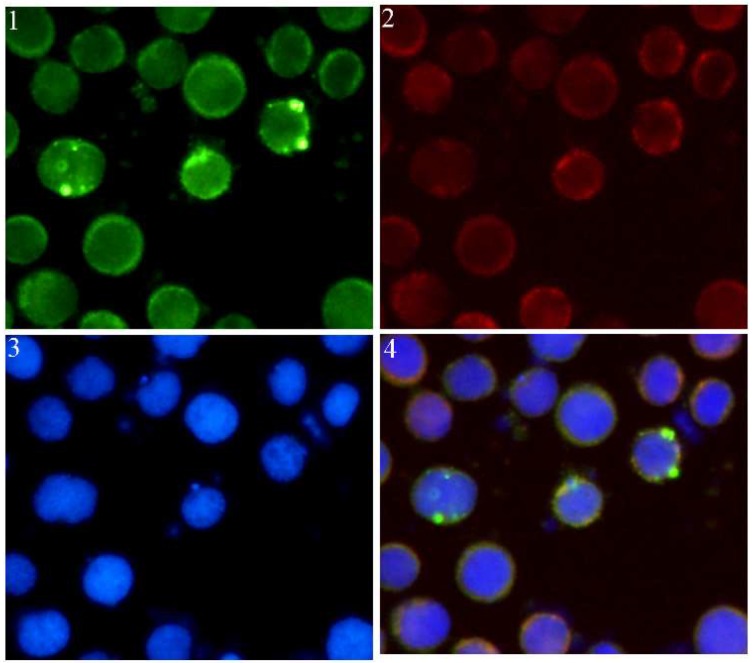
Subcellular localization of Sf-IBM1 and cytochrome c in Sf9 cells observed under a fluorescence microscopy (40×). (**1**) The green fluorescence represented the recombinant protein IBM1. (**2**) The red fluorescence indicated the distribution of cytochrome c in Sf9 cells. (**3**) The blue fluorescence showed the nuclei stained with Hoechst 33258. (**4**) The figure merged with three fluorescence.

To examine the distribution of intracellular Sf-IBM1 in Sf9 cells transfected with pIZT/V5-His-Sf-IBM1, indirect immunostaining was carried out with a polyclonal rabbit antibody against Sf-IBM1 and a polyclonal mouse antibody against cytochrome c. After 24 h of transfection of pIZT/V5-His-Sf-IBM1 in Sf9 cells, the position of the Sf-IBM1 protein shown with green fluorescence overlapped with cytochrome c, which indicated the distribution of mitochondria (Figure 5). Our results indicated that Sf-IBM1 was distributed mainly in mitochondria together with the mitochondrial protein cytochrome c.

### 2.6. The Apoptosis Induced by Sf-IBM1 Could Be Blocked by Z-VAD-FMK and Sf-IAP1

**Figure 6:**
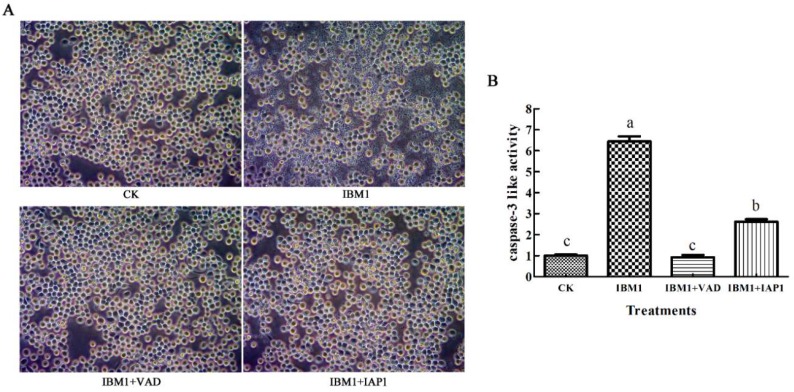
Apoptosis induced by the overexpression of Sf-IBM1 was inhibited by both carbobenzoxy-valyl-alanyl-aspartyl-[*O*-methyl]-fluoromethylketone (Z-VAD-FMK) and Sf-IAP1. (**A**) Morphological changes of cells treated with different agents by inverted phase contrast microscopy (20×). (**B**) Caspase-3 activity in cells under different treatments. CK: normal cells; IBM1: cells transfected with pIZT/V5-His-Sf-IBM1; IBM1+VAD: cells treated with Z-VAD-FMK and transfected with pIZT/V5-His-Sf-IBM1; IBM1+IAP1: cells co-transfected with pIZT/V5-His-Sf-IBM1 and pIZT/V5-His-Sf-IAP1.

To determine the pathway of apoptosis induced by Sf-IBM1, the impact of the caspase universal inhibitor Z-VAD-FMK and IAP1 on apoptosis was examined. As shown in Figure 6A, apoptosis induced by Sf-IBM1 was suppressed by Z-VAD-FMK completely, indicating that caspases were involved in the pathway of Sf-IBM1. Similarly, apoptosis induced by Sf-IBM1 was inhibited by IAP1. Further analysis indicated that there was no change in caspase-3 activity. However, caspase-3 activity increased significantly by IAP1 (Figure 6B). These results suggested that Sf-IBM1 induced apoptosis through activating the caspase-dependent apoptotic pathway, and the pathway could be blocked by the anti-apoptotic protein Sf-IAP1.

## 3. Discussion

In *Drosophila*, proteins in the RHG family function as IAP antagonists via binding to the BIR domains of IAPs, resulting in the release of active caspases as well as the promotion of ubiquitination and degradation of IAPs [24]. Five RHG family members, named Reaper, Hid, Grim, Sickle, and Jafrac2, have been found in *Drosophila*. Little sequence similarity exists among the different RHG family members except for the IAP binding motif in the N-terminus. The IAP binding motif is required for the pro-apoptotic function [13,25]. Interestingly, only one orthologue of reaper was found in Lepidopterans and named as IBM1 [22]. In this study, a reaper homolog named Sf-IBM1 was identified from a transcriptome of Sf9 cells. Consistent with IBM1s identified from other lepidopterans, Sf-IBM1 also has the highly conserved IAP binding motif, which is responsible for the pro-apoptotic function, and the GH3 motif, which regulates protein translocation to mitochondria [18,19]. Despite the sequence diversity observed between IBM1 in lepidopteran and Reaper of *Drosophila*, the pro-apoptotic function could be highly conserved because of the IAP binding motif.

As an important type of programmed cell death, apoptosis has emerged as a critical event that manifests in a large amount of tissue degradation and removes redundant cells in the metamorphosis process during egg-larva-pupa-adult stages transition in lepidopteran insects [26,27]. In *Drosophila*, the transcriptional expression of RHG family genes was considered to be the premonition of the occurrence of apoptosis [12]. In this study, *Sf-IBM1* was highly expressed in eggs, pupae, and adults, indicating that it may play a key role in promoting the occurrence of apoptosis and may drive the metamorphosis process in *S. frugiperda*. During larva-pupa transition, many organs of insects need to be degenerated and remade, including midgut, fat body, head glands, etc. [26]. The differences in expression levels of *Sf-IBM1* among different tissues could forebode the different apoptotic states associated with these tissues.

Reaper and its homologs in several other insect species have been found to be involved in apoptosis induced by adverse stimuli [12]. For example, UV irradiation could increase the expressions of Reaper, Grim, and Hid in association with the induction of apoptosis in *Drosophila* and the Caribbean fruit fly, *Anastrepha suspensa* [20,22]. Rapid induction of the Reaper ortholog *mx* in the midgut of *Aedes aegypti* was observed in mosquitoes infected with baculovirus *Cuni*NPV (*Culex nigripalpus* nucleopolyhedrovirus) [28]. The transcript levels of *Bm-IBM1* in BmN cells and pupae of *Bombyx mori* were up-regulated after infection by baculovirus *B. mori* Nucleopolyhedrovirus [13]. Sl-IBM1 was induced by azadirachtin along with increased apoptosis in the midgut of *Spodoptera litura* [29]. In this study, we demonstrated that azadirachtin, camptothecin, and UV induced expression of Sf-IBM1 in Sf9 cells along with increased apoptosis. Our results together with previous reports indicate that Sf-IBM1 plays an important role in apoptosis in insects.

The role of Reaper in apoptosis has been relatively extensively studied in *Drosophila*. As mentioned before, Reaper regulates apoptosis via the IAP binding motif and the GH3 motif [30]. The IAP binding motif ensures the pro-apoptotic function of Reaper via its interaction with the BIR domains of IAPs, resulting in acceleration of IAP degradation. The disruption of the balance between IAPs and caspases in non-apoptotic cells results in releasing caspases for cleaving other cellular substrates, eventually leading to apoptosis [24]. The GH3 domain of Reaper, on the other hand, is sufficient by itself to induce cell death in vitro [31]. The GH3 domain is required for protein localization in mitochondria via binding to lipids on the mitochondrial outer membrane [18]. During Reaper translocation from cytoplasm to mitochondria, the GH3 domain binds to the C-terminal mitochondrial targeting sequence and promotes the transfer of Reaper from cytoplasm to mitochondria, leading to the changes of mitochondrial ultrastructure, mitochondrial disruption, and the activation of mitochondrial apoptosis signaling pathway and caspases cascade, which ultimately induces apoptosis [19,32,33,34]. Transient expression of Reaper induces apoptosis in insect and mammalian cell lines [13,35,36]. In *B. mori*, the overexpression of Bm-IBM1 induced apoptosis in BmN and Sf9 cells, and it was confirmed to localize to mitochondria, which was similar to Reaper localization [13]. Our results also revealed that overexpression of Sf-IBM1 induced apoptosis in Sf9 cells, and immunofluorescence staining demonstrated that Sf-IBM1 was distributed in mitochondria.

As the regulators and the executors of apoptosis, caspases determine whether cells undergo apoptosis [37]. In this study, the overexpression of Sf-IBM1 increased the caspase-3 like activity and was blocked by caspase universal inhibitor Z-VAD-FMK. These results indicated that Sf-IBM1 activated the caspase-dependent pathway and induced apoptosis. IAPs contain the evolutionarily conserved BIR domains and the C-terminal RING finger, which display the activity of E3 ubiquitin ligase and play critical roles in apoptosis regulation by interacting with caspases and inhibiting the activities [38,39]. As is known, the RHG family proteins inhibit the function of DIAP1. Conversely, IAPs have the function to inhibit RHG counterparts and caspases [40]. In this study, apoptosis induction by over-expression of Sf-IBM1 could be blocked by simultaneous over-expression of Sf-IAP1. The results further confirmed the close interaction between IBM1 and IAP1. Together, our findings indicate that Sf-IBM1 also activates apoptosis via the mitochondrial signaling pathway and the caspase cascade.

## 4. Materials and Methods

### 4.1. Cell Culture and Insect Rearing

Sf9 cells were cultured in 35 mm^2^ flasks (Nest, Wuxi, China) with Grace’s insect cell culture medium (Gibco, Grand Island, NY, USA) containing 10% fetal bovine serum (Gibco) at 27 °C. The medium was changed every two days, and the subculture was operated after the growth of the cells to 70–80% of the flask.

A colony of *S. frugiperda* was derived from a cornfield collection in Huizhou city, Guangdong province, China. Larvae were fed with an artificial diet, while 10% honey water was used as the food for adults. The population was maintained in an incubator with the following parameters: 25 ± 1 °C, 70% relative humidity, and a photoperiod of 12:12 (L:D) h.

### 4.2. Cloning and Sequencing Sf-IBM1

A transcriptome of Sf9 cells was performed, and the putative apoptosis-related genes were identified through BLAST and KEGG orthologue annotations in our previous study [39]. For the blast research, the threshold of E-value of 1e^−5^ was used, and a sequence with 285 bp length showed the 8^e−118^ with the IBM1 from *L. dispar* (NM_001166341.1) was annotated as Sf-IBM1. To obtain the complete sequence information of Sf-IBM1, Sf9 cells were collected and cracked by RNAiso Plus (TaKaRa, Tokyo, Japan), and the total RNA of the cells was isolated according to the operational instructions. Total RNA was used to synthesize the first-strand cDNA using a PrimeScript^®^ 1st Strand cDNA Synthesis Kit (TaKaRa). Primers were designed by Primer Premier 5 and listed in Supplementary Materials Table S1. The reverse transcription was performed using TaKaRa LA Taq^®^ (TaKaRa) under the reaction conditions of 94 °C 3 min; 94 °C 30 s, 52 °C 30 s, 72 °C 30 s, 32 cycles; 72 °C 10 min. A full-length transcript was obtained by RACE. Specifically, the 3′ untranslated region was cloned using queshipcr with TaKaRa LA Taq^®^ (TaKaRa), while the 5′ untranslated region was obtained using an Advantage 2 polymerase mix in SMARTer^TM^ 5′ RACE cDNA Amplification Kit with the PCR reaction carried as the following: 94 °C 3 min; 94 °C 30 s, 68 °C 30 s, 72 °C 1 min, 32 cycles; 72 °C 10 min. PCR products were separated on an 1.2% agarose gel, and the candidate bands were purified using a Universal DNA Purification Kit (TIANGEN, Beijing, China). PCR fragments were sequenced directly. After obtaining 5′- and 3′-untranslated region, a full-length cDNA was obtained by PCR using the same primer set. ClustalW in MEGA6 software (Kyoto University Bioinformatics Center, Kyoto, Japan) was used to compare the amino acid sequences, and the bootstrap test for 1000 replicates and a Poisson model in the neighbor-joining method were used to construct the phylogenetic tree, while other parameters were set to default.

### 4.3. qRT-PCR

To investigate the expression pattern of *Sf-IBM1*, the *S. frugiperda* samples of different developmental stages including eggs, first-six instar larvae, pupae, female, and male adults were collected. Moreover, the tissues including head, cuticle, fat body, midgut, and Malpighian tubule were dissected from six instar larvae and washed with cold phosphate-buffered saline (PBS). Three replicates were conducted, and all the samples were immediately frozen in liquid nitrogen.

In addition, the expression profiles of Sf-IBM1 under apoptotic conditions were performed, and three stimuli (azadirachtin, camptothecin, and UV) that could induce apoptosis in Sf9 cells were chosen. The Sf9 cells were seeded into the 6-well plates and incubated at 27 °C overnight. The azadirachtin samples were the cells treated with azadirachtin at the concentration of 0.75 μg/mL for 12, 24, 36, and 48 h, while the camptothecin samples were the cells with 1 μg/mL camptothecin treatment for 6, 9, 12, and 24 h, respectively. In addition, the UV samples were cells treated with UV (254 nm wavelength and ≥300 Lx intensity) for 5 min and then incubated at 27 °C for 3, 6, 9, and 12 h, respectively.

qRT-PCR was performed as described previously [41]. *Sf-GAPDH* was chosen as the reference gene, and the quantitative primers were designed and are listed in Supplementary Materials Table S1. qRT-PCR was conducted on CFX Connect™ Real-Time System (Bio-Rad, Hercules, CA, USA) with 10 μL reaction system, which consisted of 5 μL SsoAdvanced™ SYBR^®^ Green Supermix (Bio-Rad), 1 μL forward primer and reverse primer, 0.5 μL template cDNA, and 2.5 μL sterilized water. The program was executed with 95 °C for 3 min, 40 cycles of 95 °C for 10 s, 60 °C for 10 s, 72 °C for 20 s, and finally followed by a dissociation step. The expression profiles of *Sf-IBM1* were calculated by the 2^−ΔΔ*C*t^ method, and the differential significance analysis was performed by One-way ANOVA and Duncan’s new multiple range test (DMRT) in SPSS17.0 (IBM SPSS, Chicago, IL, USA).

### 4.4. Western Blot

The procedure of western blot was described in detail in our previous publication [41]. The total protein samples from Sf9 cells were extracted by CytoBuster^TM^ Protein Extraction Reagent (Novagen, Kenilworth, NJ, USA), and the cytoplasmic and the mitochondrial proteins were extracted by Nuclear and Cytoplasmic Protein Extraction Kit and Mitochondrial Protein Extraction Kit (KeyGEN BioTECH, Nanjing, China) according to the manufacturer’s protocol. The Bradford method was used to detect protein concentration. In brief, 20 μg of protein from different samples was subjected to 12% SDS-PAGE gel and transferred to polyvinylidene fluoride (PVDF) membranes (Millipore, Boston, MA, USA). The membranes were blocked in tris-buffered saline (TBS) supplemented with 5% fat-free milk at 4 °C overnight and incubated with the primary antibodies anti-Sf-IBM1 and anti-GAPDH (Beyotime Biotechnology, Shanghai, China) by the dilution ratio of 1:3000 at room temperature for 2 h. Then, membranes were washed with TBS buffer supplemented with 0.05% Tween 20 (TBST) and incubated with the diluted horseradish peroxidase-conjugated secondary antibodies at room temperature for more than 2 h. The protein bands were visible by enhanced chemiluminescence (ECL) western blot detection reagents (Bio-Rad, Hercules, CA, USA) and recorded by ChemiDoc^TM^ MP imaging system (Bio-Rad).

### 4.5. Recombination Plasmid Construction

The coding regions of *Sf-IBM1* and *Sf-IAP1* were PCR-amplified with TaKaRa *LA Taq*^®^ (TaKaRa), and the primers containing restriction sites are listed in Table S1. The PCR products of *Sf-IBM1* and *Sf-IAP1* were digested with *EcoR I* and *Xba I* and introduced into the *EcoR I/Xba I* site of pIZT/V5-His, generating the recombination plasmids pIZT/V5-His-Sf-IBM1 and pIZT/V5-His-Sf-IAP1. All these recombination plasmids were constructed for transient expression of these proteins in Sf9 cells.

### 4.6. Cell Transfection and Morphological Observation

Transfection of Sf9 cells was performed by FuGENE^®^ HD Transfection Reagent (Promega, Madison, WI, USA). Monolayer cultures cells prepared in 35 mm cell culture dishes (Corning, Corning, NY, USA) and incubated overnight at 27 °C were transfected with 100 μL serum-free medium containing 2 μg recombination plasmid and 6 μL FuGENE^®^ HD according to the operating instructions. After 24 h transfection, the cell morphology was observed by an inverted phase-contrast microscope (Lecia, Frankfurt, Germany). Additionally, 20 μM Caspase universal inhibitor Z-VAD-FMK combined with pIZT/V5-His-Sf-IBM1 transfection was also performed in Sf9 cells and determined by inverted phase contrast microscope (IPCM, Olympus, Tokyo, Japan).

### 4.7. DAPI Staining

To analyze the nuclear morphological changes of Sf9 cells after they were transfected with the plasmid pIZT/V5-His-Sf-IBM1, DAPI (KeyGEN BioTECH, Nanjing, China) was selected to dye the cells. The cells after transfection were fixed and washed by 1 μg/mL DAPI methanol solution and incubated with 500 μL DAPI solution at room temperature for 15 min. Then, the solution was removed, and the cells were washed with methanol solution. Finally, the morphological changes of the Sf9 cell nucleus were observed by fluorescence microscope (Nikon, Tokyo, Japan).

### 4.8. DNA Ladder Assay

Cells under different treatments were collected by centrifugation of 10,621× *g* for 1 min, and the DNA of Sf9 cells was isolated by TIANamp Genomic DNA Kit (TIANGEN, Beijing, China) based on the provided protocol. The DNAs were then were detected on 1.0% (*w*/*v*) agarose gel and recorded by Universal Hood II ChemiDoc Molecular Imager XR+ (Bio-Rad, Hercules, CA, USA).

### 4.9. Caspase-3 Activity Assay

The Caspase-3 activity assay was measured using a Caspase-3 Colorimetric Assay Kit (KeyGEN BioTECH, Nanjing, China) as previously described [29]. Cells under different treatments were collected by centrifugation and washed with PBS for two times. Cells were lysed in a cold lysis buffer for 60 min, and the concentrations of the protein samples were determined following the Bradford method. Mixtures containing 150 mg proteins and 5 μL Caspase-3 substrate (integrating specific luminescence substrate) were incubated in the dark for 4 h at 37 °C, and the absorbance at 405 nm was detected under a microplate reader (Thermo Scientific, Waltham, MA, USA).

### 4.10. Immunofluorescence Staining

Cells under different treatments were washed with PBS three times and fixed with 4% paraformaldehyde solution for 20 min at room temperature. Then, the solution was removed, and the cells were washed again with PBS twice. PBS solution with 1% Triton-X was added and incubated for 10 min. The cells were washed with PBS twice again. PBS solution containing 1% bovine serum albumin (BSA) was used to block the cells for 1 h. Primary antibodies were then added with 1:300 dilution and were incubated for 2 h at room temperature. After washing, fluorescent secondary antibody was then added with 1:1000 dilution in PBS containing 1% BSA and incubated for another 2 h at room temperature. The cells were washed with PBS three times and then stained with Hoechst 33258 for 5 min. The cells were washed with PBS again and were observed under a fluorescence microscope (Nikon, Tokyo, Japan).

## 5. Conclusions

In summary, we cloned a gene encoding a Reaper homolog and named *Sf-IBM1* from Sf9 cells. The predicted protein conserved the IAP binding motif and the GH3 motif. Apoptotic stimuli induced the expression of Sf-IBM1 along with induced apoptosis. Transient expression of *Sf-IBM1* induced apoptosis in Sf9 cells, and Sf-IBM1 was located in mitochondria. Apoptosis induced by Sf-IBM1 was blocked by the caspase universal inhibitor Z-VAD-FMK. Co-expression of Sf-IAP also inhibited the apoptosis induced by Sf-IBM1. Our results revealed that functions of Sf-IBM1 are conserved between Reaper in *Drosophila* and IBM1 in lepidopterans. Our results provide a basis for further study of the apoptosis mechanism in *S. frugiperda*.

## Supplementary Materials

Supplementary materials can be found at https://www.mdpi.com/1422-0067/21/8/2729/s1.

## References

[1] Waldron J.A., Jones C.I., Towler B.P., Pashler A.L., Grima D.P., Hebbes S., Crossman S.H., Zabolotskaya M.V., Newbury S.F. (2015). Xrn1/Pacman affects apoptosis and regulates expression of hid and reaper. Biol. Open.

[2] Nirmala X., Schetelig M.F., Zimowska G.J., Zhou L., Handler A.M. (2015). Pro-apoptotic gene regulation and its activation by gamma-irradiation in the Caribbean fruit fly, *Anastrepha suspensa*. Apoptosis.

[3] Hwangbo D.S., Biteau B., Rath S., Kim J., Jasper H. (2016). Control of apoptosis by Drosophila DCAF12. Dev. Biol..

[4] Huang N., Civciristov S., Hawkins C.J., Clem R.J. (2013). SfDronc, an initiator caspase involved in apoptosis in the fall armyworm *Spodoptera frugiperda*. Insect Biochem. Mol. Biol..

[5] Tajbakhsh A., Kovanen P.T., Rezaee M., Banach M., Moallem S.A., Sahebkar A. (2020). Regulation of efferocytosis by caspase-dependent apoptotic cell death in atherosclerosis. Int. J. Biochem. Cell Biol..

[6] Yuan S., Akey C.W. (2013). Apoptosome structure, assembly, and procaspase activation. Structure.

[7] Kang Y., Neuman S.D., Bashirullah A. (2017). Tango7 regulates cortical activity of caspases during reaper-triggered changes in tissue elasticity. Nat. Commun..

[8] Ito H., Bando H., Shimada T., Katsuma S. (2014). The BIR and BIR-like domains of *Bombyx mori* nucleopolyhedrovirus IAP2 protein are required for efficient viral propagation. Biochem. Biophys. Res. Commun..

[9] Hamajima R., Iwamoto A., Tomizaki M., Suganuma I., Kitaguchi K., Kobayashi M., Yamada H., Ikeda M. (2016). Functional analysis of inhibitor of apoptosis 1 of the silkworm *Bombyx mori*. Insect Biochem, Mol. Biol..

[10] Lee T.V., Fan Y., Wang S., Srivastava M., Broemer M., Meier P., Bergmann A. (2011). Drosophila IAP1-mediated ubiquitylation controls activation of the initiator caspase DRONC independent of protein degradation. PLoS Genet..

[11] Kamezaki M., Yokoi K., Miurarna K. (2018). Interference mediated knockdown of an inhibitor of apoptosis protein induces apoptosis in *Mythimna separata* (Lepidoptera: Noctuidae). Eur. J. Entomol..

[12] Vasudevan D., Ryoo H.D. (2015). Regulation of Cell Death by IAPs and their Antagonists. Curr. Top. Dev. Biol..

[13] Wu Y., Wu Y., Hui T., Wu H., Wu Y., Wang W. (2013). Reaper homologue IBM1 in silkworm *Bombyx mori* induces apoptosis upon baculovirus infection. FEBS Lett..

[14] Wolf B.B., Green D.R. (2002). Apoptosis: Letting slip the dogs of war. Curr. Biol..

[15] Wang H., Clem R.J. (2011). The role of IAP antagonist proteins in the core apoptosis pathway of the mosquito disease vector *Aedes aegypti*. Apoptosis.

[16] Yoo S.J., Huh J.R., Muro I., Yu H., Wang L., Wang S.L., Feldman R.M.R., Clem R.J., Muüller H.A.J., Hay B.A. (2002). Hid, Rpr and Grim negatively regulate DIAP1 levels through distinct mechanisms. Nat. Cell Biol..

[17] Zachariou A., Tenev T., Goyal L., Agapite J., Steller H., Meier P. (2003). IAP-antagonists exhibit nonredundant modes of action through differential DIAP1 binding. EMBO J..

[18] Freel C.D., Richardson D.A., Thomenius M.J., Gan E.C., Horn S.R., Olson M.R., Kornbluth S. (2008). Mitochondrial localization of Reaper to promote inhibitors of apoptosis protein degradation conferred by GH3 domain-lipid interactions. J. Biol. Chem..

[19] Sandu C., Ryoo H.D., Steller H. (2010). Drosophila IAP antagonists form multimeric complexes to promote cell death. J. Cell Biol..

[20] Liu B., Becnel J.J., Zhang Y., Zhou L. (2011). Induction of reaper ortholog mx in mosquito midgut cells following baculovirus infection. Cell Death Differ..

[21] Schetelig M.F., Nirmala X., Handler A.M. (2011). Pro-apoptotic cell death genes, hid and reaper, from the tephritid pest species, *Anastrepha suspensa*. Apoptosis.

[22] Bryant B., Zhang Y., Zhang C., Santos C.P., Clem R.J., Zhou L. (2009). A lepidopteran orthologue of reaper reveals functional conservation and evolution of IAP antagonists. Insect Mol. Biol..

[23] Huang H., Joazeiro C.A., Bonfoco E., Kamada S., Leverson J.D., Hunter T. (2000). The inhibitor of apoptosis, cIAP2, functions as a ubiquitin-protein ligase and promotes in vitro monoubiquitination of caspases 3 and 7. J. Biol. Chem..

[24] Clavier A., Rincheval-Arnold A., Colin J., Mignotte B., Guénal I. (2016). Apoptosis in Drosophila: Which role for mitochondria?. Apoptosis.

[25] Zhang J.Y., Pan M.H., Sun Z.Y., Huang S.J., Yu Z.S., Liu D., Zhao D.H., Lu C. (2010). The genomic underpinnings of apoptosis in the silkworm, *Bombyx mori*. BMC Genom..

[26] Tettamanti G., Casartelli M. (2019). Cell death during complete metamorphosis. Philos. Trans. R. Soc. Lond. B Biol. Sci..

[27] Bao X., Chen P., Liu T., Wang L., Liu W., Pan M., Lu C. (2017). Advances in apoptosis-related genes in the silkworm, *Bombyx mori*. Acta Entomol. Sin..

[28] Ying Q.Q., Liu T., Gu W. (2012). Effect of ultraviolet irradiation on the expressions of apoptosis related genes reaper, grim, hid in *Drosophila*. Chin. J. Gerontol..

[29] Shu B., Zhang J., Cui G., Sun R., Yi X., Zhong G. (2018). Azadirachtin affects the growth of *Spodoptera litura* Fabricius by inducing apoptosis in larval midgut. Front. Physiol..

[30] Thomenius M., Kornbluth S. (2006). Multifunctional reaper: Sixty-five amino acids of fury. Cell Death Differ..

[31] Claveria C., Caminero E., Martinez A.C., Campuzano S., Torres M. (2002). GH3, a novel proapoptotic domain in Drosophila Grim, promotes a mitochondrial death pathway. EMBO J..

[32] Haining W.N., Carboy-Newcomb C., Wei C.L., Steller H. (1999). The proapoptotic function of Drosophila Hid is conserved in mammalian cells. Proc. Natl. Acad. Sci. USA.

[33] Abdelwahid E., Yokokura T., Krieser R.J., Balasundaram S., Fowle W.H., White K. (2007). Mitochondrial disruption in Drosophila apoptosis. Dev. Cell.

[34] Morishita J., Kang M.J., Fidelin K., Ryoo H.D. (2013). CDK7 regulates the mitochondrial localization of a tail-anchored proapoptotic protein, Hid. Cell Rep..

[35] Vucic D., Seshagiri S., Miller L.K. (1997). Characterization of reaper- and FADD-induced apoptosis in a lepidopteran cell line. Mol. Cell Biol..

[36] Tait S.W., Werner A.B., de Vries E., Borst J. (2004). Mechanism of action of Drosophila Reaper in mammalian cells: Reaper globally inhibits protein synthesis and induces apoptosis independent of mitochondrial permeability. Cell Death Differ..

[37] Yu H., Li Z.Q., Ou-Yang Y.Y., Huang G.H. (2019). Identification of four caspase genes from *Spodoptera exigua* (Lepidoptera: Noctuidae) and their regulations towards different apoptotic stimulations. Insect Sci..

[38] Chai J.J., Yan N., Huh J.R., Wu J.W., Li W.Y., Hay B.A., Shi Y.G. (2003). Molecular mechanism of Reaper-Grim-Hid mediated suppression of DIAP1-dependent Dronc ubiquitination. Nat. Struct. Biol..

[39] Shu B., Zhang J., Sethuraman V., Cui G., Yi X., Zhong G. (2017). Transcriptome analysis of *Spodoptera frugiperda* Sf9 cells reveals putative apoptosis-related genes and a preliminary apoptosis mechanism induced by azadirachtin. Sci. Rep..

[40] Betz A., Ryoo H.D., Steller H., Darnell J.E. (2008). STAT92E is a positive regulator of Drosophila inhibitor of apoptosis 1 (DIAP/1) and protects against radiation-induced apoptosis. Proc. Natl. Acad. Sci. USA.

[41] Shu B., Jia J., Zhang J., Sethuraman V., Yi X., Zhong G. (2018). DnaJ homolog subfamily A member1 (DnaJ1) is a newly discovered anti-apoptotic protein regulated by azadirachtin in Sf9 cells. BMC Genom..

