# Supplementary material for: Pro-Apoptotic Function Analysis of the Reaper Homologue IBM1 in Spodoptera frugiperda
Source: Int J Mol Sci. 2020 Apr 15;21(8):2729. doi: 10.3390/ijms21082729 (PMC7215429; doi:10.3390/ijms21082729)
Supplement: Supplementary file 1 [file ijms-21-02729-s001.pdf]

Supplemental Table 1. The information of primers used in this study.

Primer name	Primer sequences
Sf-IBM1-cds-F	ATGGCTATAGCATTCAACTT
Sf-IBM1-cds-R	TTATCTGATGGTATTGAAGAGAGA
Sf-IBM1-3’-F1	GCAAGAAGTCCAGGAGAATA
Sf-IBM1-3’-F2	CGACCTACATCGTCAACCTA
Sf-IBM1-5’-R1	AGGCTGTCGTGAGACCAAGATGTG
Sf-IBM1-5’-R2	GCGAGGGAGACCTTTATGATGGC
Sf-IBM1-RT-F	TCCAGGAGAATAGGCGAGGTG
Sf-IBM1-RT-R	CGAGGGAGACCTTTATGATGGC
Sf-GAPDH-RT-F	TTGACGGACCCTCTGGAAAA
Sf-GAPDH-RT-R	ACGTTAGCAACGGGAACACG
Sf-IBM1-F(*EcoR I*)	CGGGAATTCGCCACCATGGCTATAGCATTCAACTT
Sf-IBM1-R(*Xba I*)	GCTCTAGATCTGATGGTATTGAAGAGAGA
Sf-IAP1-F(*EcoR I*)	CGGGAATTCGCCACCATGTGGTCGTGTTCCTTACCT
Sf-IAP1-R(*Xba I*)	GCTCTAGACGAGAAATATAACCGCACTGC

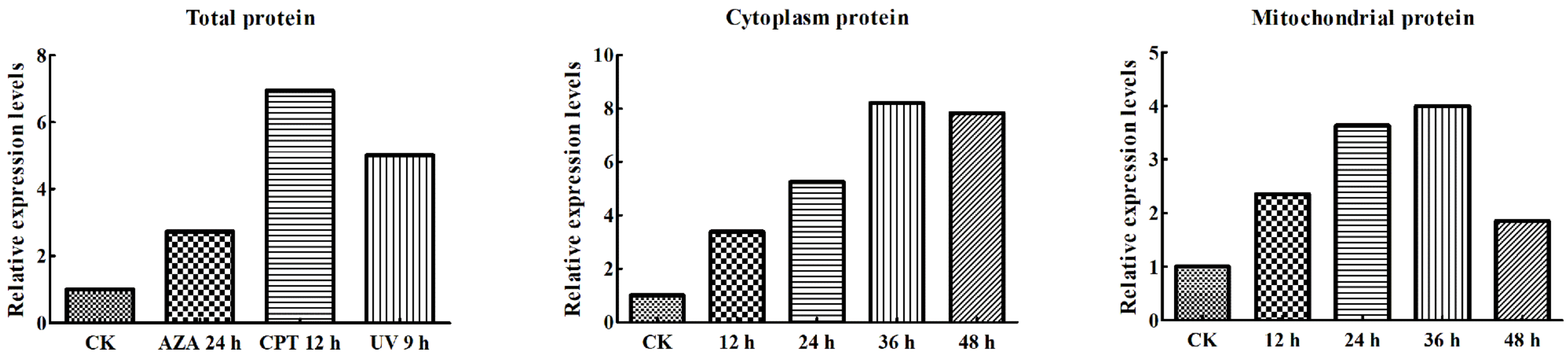

Supplemental Figure 1. Quantitative analysis of Sf-IBM1 protein expression detected by western blot in Figure 3. Fig S1A: The quantitative analysis results of Sf-IBM1expression in Sf9 cells with different treatments. Fig S1B: The relative expression values of Sf-IBM1 in cytoplasm protein of Sf9 cells with azadirachtin treatment for different times. Fig S1C: The relative expression values of Sf-IBM1 in the mitochondrial protein of Sf9 cells treated with azadirachtin for different times.
